# Deficits of perceived spatial separation induced prepulse inhibition in patients with schizophrenia: relationships to symptoms and neurocognition

**DOI:** 10.1186/s12888-017-1276-4

**Published:** 2017-04-11

**Authors:** Ning-Bo Yang, Qing Tian, Yu Fan, Qi-Jing Bo, Liang Zhang, Liang Li, Chuan-Yue Wang

**Affiliations:** 1 Department of Psychiatry, Beijing Anding Hospital, Capital Medical University, No.5 Ankang Lane, Dewai Avenue, Xicheng District, Beijing, 100088 China; 2 Beijing Key Laboratory of Mental Disorders, No.5 Ankang Lane, Dewai Avenue, Xicheng District, Beijing, 100088 China; 3 Beijing Institute for Brain Disorders Center of Schizophrenia, No.5 Ankang Lane, Dewai Avenue, Xicheng District, Beijing, 100088 China; 4 Department of Psychology, Peking University, Beijing, 100871 China; 5 Key Laboratory on Machine Perception (Ministry of Education), Beijing, 100871 China; 6 McGovern Institute for Brain Research, Beijing, 100871 China

**Keywords:** Schizophrenia, Prepulse inhibition, Perceived spatial separation, Clinical symptom, Thought disorder, Cognitive function, Attentional function

## Abstract

**Background:**

Prepulse inhibition (PPI) and attention were impaired, which may cause psychotic symptoms and (or) hinder the cognitive functions in schizophrenia. However, due to the measurement methods of PPI, findings about the relationship between PPI and clinical symptoms, cognitive performances have been equivocal.

**Methods:**

Seventy-five schizophrenia patients (SZ) and 50 healthy controls (HC) were assessed in a modified acoustic PPI paradigm, named perceived spatial separation-induced PPI (PSS-PPI), compared to perceived spatial co-location PPI (PSC-PPI) with inter-stimulus interval (ISI) of 120 ms. Repeatable Battery for the Assessment of Neuropsychological Status and the Stroop Color-Word Test were administered to all subjects.

**Results:**

Significant decrease in the modified PPI was found in the patients as compared to the controls, and effect sizes (Cohen’*d*) for patients vs. HCs % PPI levels achieved a significant level (PSC-PPI *d* = 0.84, PSS-PPI *d* = 1.27). A logistic regression model based on PSS-PPI significantly represented the diagnostic grouping (χ^2^_=_ 29.3; *p* < 0 .001), with 85.2% area under ROC curve in predicting group membership.

In addition, patients exhibited deficits in neurocognition. Among patients of “non-remission”, after controlling for gender, age, education, duration, recurrence times, onset age, cigarettes per day and chlorpromazine equivalent dosage, PSS-PPI levels were associated with positive and negative symptoms, PANSS total and thought disorder (P1, P6, P7, N5, N7, G9). In multiple linear regression analyses, male and higher attention scores contributed to better PSC-PPI and PSS-PPI in controls group, while larger amount of smoke and longer word-color interfere time contributed to poor PSS-PPI. In patients’ group, higher education and attention scores contributed to better PSS-PPI, while repeated relapse contributed to poor PSS-PPI.

**Conclusions:**

The acoustic perceived spatial separation-induced PPIs may bring to light the psychopathological symptoms, especially for thought disorder, and the mechanism(s) of the novel PPI paradigm was associated with attention function.

## Background

Abnormalities of information-processing are viewed as core elements of the cognitive deficits and emotion disturbances that characterize schizophrenia [1, 2]. A major feature of information processing deficits in schizophrenic patients is deficient sensorimotor gating, which can be examined operational via the use of prepulse inhibition (PPI) and P50 [3, 4].

Prepulse inhibition occurs when a non-startling stimulus (the prepulse) results in a reduced startle response to a subsequent startling stimulus [5]. Recently, PPI has been shown to be reduced in schizophrenic patients as well as in unaffected relatives [6], and be considered a promising candidate endophenotype for schizophrenia [7, 8].

Most previous studies used “passive” paradigm to investigate prepulse inhibition, while, more and more evidence has shown that individuals who direct their attention to the prepulse signal show enhanced PPI [9–11]. Importantly, paying attention to a prepulse that is presented shortly before the startle stimulus enhances PPI in normal people, but not in schizophrenia patients [12, 13]. In the active paradigm (also known as the “attention to prepulse” paradigm), most studies have demonstrated that the attentional modulation effect on PPI occurs with a lead time of 120 ms, and the same effect is not seen if the lead time is shorter (60 ms) or longer (240 ms) [13, 14]. In addition, prepulse-to-pulse intervals of 30–240 ms are typically utilized in human PPI experiments, with maximal amplitude inhibition generally occurring within intervals of approximately 120 ms [15]. Moreover, when a noise masker is presented in the active paradigm, a precedence-effect-based perceived separation is introduced between the noise masker and the prepulse, and this introduction leads to further enhancement of PPI in rats [16].

The question can then be asked: what is the precedence effect? In a reverberant environment, listeners have the ability to integrate direct sound waves with the reflections from a sound source: attributes of the delayed and correlated reflection are captured by the direct wave, leading to a single fused image whose perceived point of origin is around the location of the leading source [17]. In humans, when both the target sound and the masker are presented by each of the two spatially separated loudspeakers with an inter-loudspeaker delay of 3 ms, recognizing the target speech under the condition of perceived target-masker spatial separation (when the leading loudspeaker was different between target and masker) is significantly better than that under the condition of perceived co-location (when the leading loudspeaker was the same for both target and masker) [18]. The enhancement of recognition is caused by higher-order processes including the improvement of selective attention to the target.

The perceived spatial separation paradigm based on the precedence effect between the target and masking signals has been previously shown to promote listener’s selective spatial attention to the target signal without changing the signal spectrum, intensity, and/or density of the audio-image. Ultimately, this improves recognition of the target signal [19, 20]. This modified PPI paradigm has been used in many animal experiments, and its effect, validity and reliability have all been reliably demonstrated [21].

The evidences that deficit in prepulse inhibition contributes to symptomatic, cognitive impairment in schizophrenia are less consistent. Studies report weak or no significant correlations between PPI and psychiatric symptom severity in schizophrenia [22–25]. Some evidence associates deficient PPI to the core schizophrenia trait, rather than symptoms, or cognitive and social functional impairment [26, 27]. Others report deficient PPI only in significant symptomatic patients [28–30]. In addition, schizophrenia patients showed impairment not only in baseline PPI but also in the attentional modulation of PPI. More importantly, the deficit of PPI in schizophrenia was more related with the symptom severity when the prepulse was attended, but not when ignored [12].

Attentional impairments may disrupt many other cognitive functions and meta-analysis studies suggest moderate-to-severe attentional impairments in schizophrenic patients [31, 32]. In line with that, recent studies in rat-based model of schizophrenia-relevant symptom have shown that PPI is a positive predictor of spatial working memory and latent inhibition [33, 34]. Furthermore, previous studies have also shown dysfunctions in selective spatial attention in schizophrenia [35]. Given this, we hypothesized that the perceived spatial separation paradigm of PPI would be a more robust impairment index and might be a more sensitive measure to specific cognitive variables that are important in schizophrenia.

In the present study, a modified PPI was conducted in a sample of Chinese schizophrenia patients to test the hypothesis that PPI deficits in patients with schizophrenia are significantly related to measures of symptom severity, neurocognitive performance. This study might provide a novel PPI paradigm for clinical research, as well as further illustration of the novel PPI paradigm.

## Methods

### Study population

Eighty-seven patients and 60 healthy controls were enrolled with right-handed and did not show any pure-tone hearing impairment at the frequency of 1000 Hz in each ear. Diagnosis of schizophrenia was confirmed by administering the Structured Clinical Interview for DSM-IV (SCID) by research psychiatrists. Enrollment criteria for schizophrenia patients included: 1) clinically stable subjects without neurologic diseases or known history of head trauma, 2) no electroconvulsive therapy within the past 6 months, and 3) no history of drug or substance abuse and dependence (except tobacco). Patients were excluded if they had unstable medical conditions or IQ below 70. All patients received antipsychotic medications as usual during the period of this study.

The control group included mentally stable, healthy individuals. All control subjects answered semi-structured questions to exclude participants with a history of drug or alcohol abuse or dependence within 6 months before testing, major head trauma involving loss of consciousness for more than 5 min, epilepsy or other neurological dysfunction or first-degree family history of psychosis. Following a detailed description of the procedures involved and overall aims of this study, all participants provided informed written consent. This study was reviewed and approved by the Independent Ethics Committee (IEC) of the Beijing Anding Hospital, Capital Medical University, China.

### Demographic details and clinical characteristics

Basic socio-demographic characteristics and clinical data were collected by a questionnaire specifically designed for this study. The psychopathology of each patients was evaluated by the Positive and Negative Syndrome Scale (PANSS, Chinese version) [36]. Patients and healthy controls were additionally assessed on Repeatable Battery for the Assessment of Neuropsychological Status (RBANS, Chinese version) [37] and the traditional card version of the Stroop Color-Word Test (Chinese version) [38]. There was good agreement on ratings performed by physicians and trained psychologists (Cohen’s = 0.80).

### Startle response measurements

**Fig. 1:**
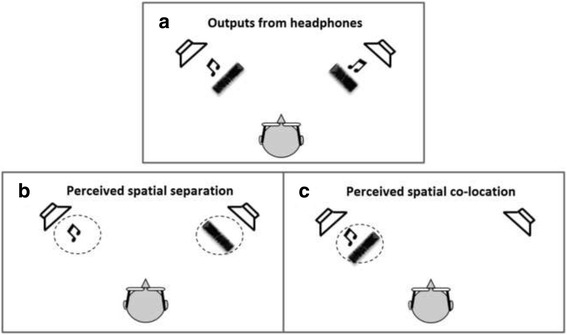
Diagrams showing the physical (Panel **a**) and perceived (Panels **b** and **c**) spatial relationship between the prepulse (the music note) and the noise masker (noise waveform). (Panel **a**) Both the prepulse and the noise masker were delivered by each of the two horizontally separated headphones. (Panel **b**) The onset of the prepulse delivered by the left headphone led that from the right headphone by 3 ms, and the image of the prepulse (music note in the circle) was perceived as coming from the left headphone; the onset of the masker from the right headphone led that from the left headphone by 3 ms, and the image of the masker (noise waveform in circle) was perceived as coming from the right headphone. Thus, the prepulse and the noise masker were perceived spatially separated. (Panel **c**) Both the onset of the prepulse and the onset of the masker presented from the left headphone led those from the right headphone, and the image of the prepulse (music note in circle) and that of the masker (noise waveform in circle) were perceived spatially co-located

Each subject sat comfortably in a recliner chair in a sound-attenuated room. Two 4 mm Ag/AgCl electrodes were positioned below and lateral to the right eye, directly over the orbicularis oculi. A ground electrode was then placed behind the right ear. Electrode resistances were <5 kΩ. The eye blink component of the acoustic startle system was evaluated using a human electromyography (EMG) startle reflex system (EMG XEYE human Startle Reflex, Tian Ming Hong Yuan Instruments Company, Beijing, China). This system recorded 300, 1-ms epochs for digitization and analysis, beginning with the startle stimulus onset. In addition, EMG activity was band-pass filtered (100-1000 Hz) and amplified by 10,000. Acoustic startle stimuli were presented binaurally through two headphones (HD 265 linear, SENNHEISER, Germany) (Fig. 1a). Acoustic signals were calibrated by a sound-level meter (AUDit and System 824, Larson Davis, USA).

**Fig. 2:**
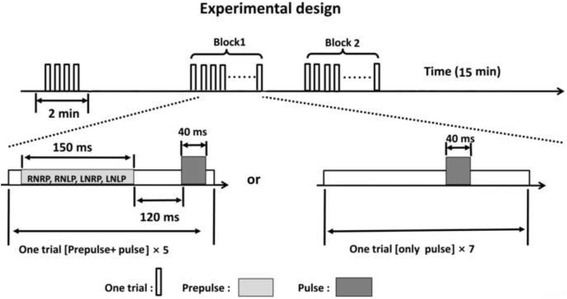
Schematic illustration of the perceived spatial separation paradigm applied in this study. In a block design (7 min), a background wideband noise was continuously delivered as the masker (left leading or right leading), 7 trials contained the startling (pulse) sound alone, and 20 trials contained the prepulse (left leading or right leading) 60 ms or 120 ms preceding the startling (pulse) noise. Trials in each block were presented randomly with the inter-trial interval about 20 s Note: RNRP (RNLP): right leading masking with prepulse co-location (separation); LNLP (LNRP): left leading masking with prepulse co-location (separation)

The test began with a 2-min adaptation period of 60 dB SPL broadband background noise, during which 4 startling sounds (broadband white noise, 40 ms, 104 dB SPL) were presented. Next, we conducted 2-blocks of PPI testing. In each block, 7 trials contained the startling sound alone delivered by two headphones, as well as 20 trials containing the prepulse (broadband white noise, 150 ms, 65 dB SPL) preceding the startling noise (120 ms between the prepulse offset and the startling-sound onset) (Fig. 2). The prepulse was presented from each headphone with the inter-loudspeaker onset delay being either +3 ms (left leading) or −3 ms (right leading). Due to the precedence effect, a single perceptually fused image of the prepulse would be perceived at the left loudspeaker location in the first block (when the left loudspeaker led) and at the right loudspeaker location in the second block (when the right loudspeaker led) [17]. In addition to the prepulse, background, wideband noise (0–10 kHz, 60 dB SPL) was continuously delivered from each of the two headphones as a masker. The inter-headphone onset delay for the masker was also either +3 or −3 ms, leading to a perceptually-fused, continuous noise-masker image that was presented either through the left headphone in one block or through the right in the other block. Thus, there were 4 (2×2) combinations of perceived locations between the prepulse and masker images across the two experimental blocks. Two types of perceived spatial relations between the prepulse and masker were created: perceptual separation (when the prepulse and masker came from a different leading headphone, Fig. 1b) and perceptual co-location (when the prepulse and masker shared the same leading headphone, Fig. 1c). Trials in each block were presented randomly with the inter-trial interval of approximately 20s (varying between 15 s and 25 s).

Startle eyeblink responses were recorded as electromyographic activity. Each trial was visually inspected for spontaneous and voluntary blinks. Trials with artifacts were excluded from analysis (total trial exclusion <4%). Electromyographic activity under either the perceived spatial co-location (PSC) or perceived spatial separation (PSS) conditions were recorded.

Percent habituation (100 × [average of first habituation block − average of second habituation block]/average of first habituation block) was calculated for each subject. PPI% = 100×(1 − [mean magnitude on prepulse trials/mean magnitude on pulse alone trials]).

### Statistical analysis

Data analysis was performed by R Development Environment and R Studio Desktop (Open Source Edition) [39]. The normality of the distributions for continuous variables was checked using a one-sample Kolmogorov–Smirnov test. Comparisons between the two groups of subjects about socio-demographic characteristics and mean scores on PPI and cognitive tests were performed using independent sample t-test and chi-square tests. Binomial logistic regression was applied for discriminating patients from controls. Associations of performance on PPI tasks with socio-demographic and clinical characteristics and other neurocognitive tests were analyzed using Pearson correlation analysis if the data followed a normal distribution; otherwise, Spearman rank correlation analysis was performed. Stepwise multiple linear regression analyses were used to identify factors that were independently associated with performance on the two types of PPI. In the stepwise regression analyses, the perceived spatial co-location PPI (PSC-PPI) and perceived spatial separation PPI (PSS-PPI) scores were entered as the dependent variable, and all variables were entered as independent variables. Two tailed tests were used in all analyses with the significance level set at 0.05.

## Results

### Subject demographics

A total of 22 participants (12 patients and 10 control subjects) were excluded for their failing to blink to the startling stimulus. Demographic characters as well as the medication status for the remaining participants are summarized in Table 1. There were no significant differences in age, years of education, smoking status and gender between the two groups. Medication status revealed that patients had the following pharmacotherapy regimens: typical antipsychotics:18.7% (*n* = 14); atypical antipsychotics: 53.3% (*n* = 40); and combination: 18% (*n* = 21), respectively.

**Table 1:** Demographic and clinical characteristics for SZ patients and HC

Variable	HC	SZ	*t/χ2*	*p*
	*N* = 50	*N* = 75		
Gender (M/F)	38/12	54/21	0.25	0.68^**a**^
Smoke				
Ratio	68.0%	60.0%	0.83	0.45^**a**^
Cigarettes per day	6.6 ± 8.8	7.3 ± 8.6	0.45	0.65^**b**^
Age (year)	42.9 ± 8.0	44.9 ± 6.7	1.57	0.12^**b**^
Education (year)	11.6 ± 3.2	10.8 ± 2.6	0.02	0.89^**b**^
Age of onset (year)		24.8 ± 6.7		
Recurrence times		4.7 ± 2.5		
Duration (year)		19.5 ± 8.4		
Medication				
FGA, n (%)		14 (18.7)		
SGA, n (%)		40 (53.3)		
FGA + SGA, n (%)		21 (18.0)		
CPZ(mg/day)		300.6 ± 247.0		
PANSS score				
Total		62.3 ± 13.5		
Positive		12.6 ± 4.7		
Negative		19.7 ± 6.5		
General		29.9 ± 5.7		

Note: Data are expressed as mean ± SD, *SD* standard deviation, *SZ* schizophrenia patients, *HC* healthy controls, *CPZ* chlorpromazine equivalent dosage

PANSS: positive and negative syndrome scale; ^a^ indicates *p* value for chi-square test; ^b^ indicates *p* value for independent sample t-test.

### Group differences in modified startle reflex and cognitive functioning

**Fig. 3:**
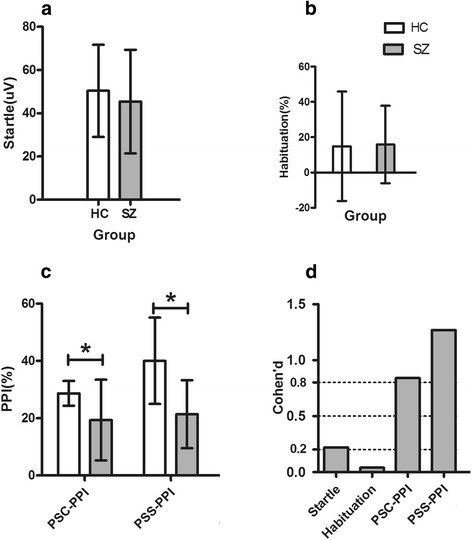
Profile analysis for the attention-modulated auditory startle reflex between HC and SZ group. Note: Data are expressed as mean ± SD; **a** Startle amplitude comparison between SZ and HC subjects; **b** Habituation comparison between SZ and HC subjects; **c** Comparison of the perceived spatial separation-induced PPI between the two groups; **d** Effect size of modified startle reflex. SZ: schizophrenia patients; HC: healthy controls; PSC-PPI: perceived spatial co-location PPI; PSS-PPI: perceived spatial separation PPI. * indicates *p* < 0.05 for independent sample t-test

Results from between-group differences on attention modulated auditory startle reflex are shown in Table 2 (Fig. 3.) Startle magnitude and habituation revealed no differences across groups. In addition, we detected significant differences between healthy control and patients for PSC-PPI and PSS-PPI. Effect sizes of the two PPI values were all large (Cohen’s d: PSC-PPI = 0.84, PSS-PPI = 1.27). The main effect of perceived spatial separation in relation to PPI was significant in healthy controls (*t* = − 10.57, *p* < 0.01), but not in schizophrenics (*t* = − 0.47, *p* = 0.64).

**Table 2:** Between-group differences in modified PPI

Variable	HC	SZ	*t*	*p* ^*a*^	*Cohen’s d*
	*N* = 50	*N* = 75			
Startle					
Mean (±SD)	50.4 ± 21.3	45.4 ± 24.0	1.18	0.24	0.22
Median (range)	47.5(14.7-81.9)	42.9(8.2-111.7)			
Habituation					
Mean (±SD)	14.9 ± 31.0	15.9 ± 22.0	0.24	0.84	0.04
Median (range)	15.8(−13.2-44.7)	16.6(−20.6-52.4)			
PSC-PPI					
Mean (±SD)	31.7 ± 25.6	9.4 ± 29.3	4.38	0.00	0.84
Median (range)	36.2(−32.7-77.8)	12.9(−5.9-70.8)			
PSS-PPI					
Mean (±SD)	50.7 ± 29.4	13.0 ± 29.8	6.97	0.00	1.27
Median (range)	48.6(−6.3-91.1)	17.29(−69.6-73.5)			

Note: Data are expressed as mean ± SD, *SD* standard deviation, *SZ* schizophrenia patients, *HC* healthy controls, *PPI* prepulse inhibition, *PSC-PPI* perceived spatial co-location PPI, *PSS-PPI* perceived spatial separation PPI; ^a^ indicates *p* value for independent sample t-test

The univariate logistic regression was used to generate models that maximally separated schizophrenia patients and healthy controls. Several parameters from Table 3 were used to evaluate the model. The goodness-of-fit test (model χ^2^: 29.3), and effect size estimate (Nagelkerke’s R^2^: 0.46) all indicated the PSS-PPI model’s greater appropriateness. The AUC of PSS-PPI obtained boosts from 72% to 85.2% for statistical significances comparing to that of PSC-PPI.

**Table 3:** Logistic regression model for different PPI distinguishing schizophrenia patients from healthy controls (ISI = 120 ms)

Predictor	χ^2^	*df*	*p*	*OR*	95% CI	*R* ^*2*^	Sensitivity	Specificity	Accuracy	PPV	NPV	AUC
PSC-PPI	14.6	1	0.00	0.97	0.95-0.98	0.17	75.0	52.0	72.0	72.7	70.3	72.0
PSS-PPI	29.3	1	0.00	0.94	0.93-0.96	0.46	87.0	66.0	78.4	79.3	76.7	85.2*

Note: The goodness-of-fit (χ^2^) and effect size indicator (Nagelkerke’s R^2^) measure how well the model represents the diagnostic groupings. *ISI* inter-stimulus interval, *PPI* prepulse inhibition, *PSC-PPI* perceived spatial co-location PPI, *PSS-PPI* perceived spatial separation PPI, *PPV* positive predictive value, *NPV* negative predictive value, *AUC* area under ROC curve; * indicates *p* < 0.05 for DeLong’s test

**Fig. 4:**
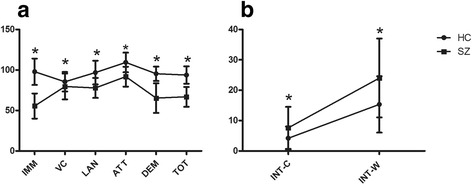
Profile analysis for cognitive domains of SZ patients and HC. Note: Data are expressed as mean ± SD. SZ: schizophrenia patients; HC: healthy controls. **a** RBAN comparison, IMM: immediate memory score; VC: visuospatial/constructional score, LAN: language score; ATT: attention score; DEM: delayed memory score; TOT: total cognitive functional score. **b** Comparison of Stroop task, INT-C: color interference time; INT-W: word interference time. * indicates *p* < 0.05 for independent sample t-test

Between-group differences on cognitive variables are shown in Table 4 (Fig. 4). Patients showed significantly poorer performance than healthy controls on all cognitive tests (all values were significant at *p* < 0.05).

**Table 4:** Between-group differences in cognitive variables

Variable	HC	SZ	*t*	*p* ^*a*^
	*N* = 50	*N* = 75		
RBANS				
IMM				
Mean(±SD)	97.9 ± 16.3	55.5 ± 15.5	14.69	0.00
Median (range)	101.5(69-123)	49(40-106)		
VC				
Mean (±SD)	85.6 ± 12.0	79.7 ± 15.9	2.23	0.03
Median (range)	87(64-121)	78(50-109)		
LAN				
Mean (±SD)	96.9 ± 14.6	77.9 ± 12.3	7.89	0.00
Median (range)	91(78-130)	76(44-105)		
ATT				
Mean (±SD)	109.4 ± 12.2	91.8 ± 12.2	7.94	0.00
Median (range)	109(88-135)	91(56-138)		
DEM				
Mean (±SD)	95.4 ± 8.9	65.3 ± 18.3	12.25	0.00
Median (range)	97(77-100)	64(18-100)		
TOT				
Mean (±SD)	93.8 ± 10.8	66.9 ± 12.2	12.62	0.00
Median (range)	91(75-122)	65(43-100)		
Stroop Test				
INT-C				
Mean (±SD)	4.2 ± 3.8	7.6 ± 6.9	2.10	0.04
Median (range)	5.1(−2.4-32.0)	1.6(−11.0-39.3)		
INT-W				
Mean (±SD)	15.3 ± 11.6	24.0 ± 13.0	4.14	0.00
Median (range)	16.1(−18.3-49.0)	22.3(−12.4-63.4)		

Note: Data are expressed as mean ± SD, *SD* standard deviation, *SZ* schizophrenia patients, *HC* healthy controls, *IMM* immediate memory score, *VC* visuospatial/constructional score, *LAN* language score, *ATT* attention score, *DEM* delayed memory score, *TOT* total composite score, *INT-C* color interference time, *INT-W* word interference time. ^a^ indicates *p* value for independent sample t-test

### Correlation between PPI and symptoms and neurocognitions

Table 5 presents the relationships between PPI and sociodemographic variables, other cognitive tests, and clinical variables. In the HC group, gender, higher scores on the attention score (ATT) and total composite score (TOT) were significantly associated with better PPI performance, and higher color interference time (INT-C) scores were associated with reflex startle, whereas less word interference time (INT-W) associated with better PSS-PPI performance. No variable was associated with any PPI tasks in patients except PANSS General negatively correlated with PSS-PPI.

**Table 5:** Correlation between modified PPI and demographic, clinical characteristics and neurocognition tasks in patients and controls

Variables	HC			SZ		
	startle	PSC-PPI	PSS-PPI	startle	PSC-PPI	PSS-PPI
Gender	0.19	0.38*	0.39*	−0.15	−0.23	−0.17
Age	0.02	0.25	0.21	0.11	0.09	0.11
Education	−0.01	0.11	−0.11	−0.08	0.05	0.08
Cigarettes per day	−0.03	−0.06	−0.03	−0.13	−0.15	−0.06
Duration				0.09	0.14	0.13
Recurrence				0.13	−0.13	−0.06
Onset age				−0.10	−0.01	−0.08
CPZe				−0.07	−0.06	-0.10
PANSS Positive				−0.13	−0.16	-0.15
PANSS Negative				−0.13	−0.05	-0.09
PANSS General				−0.03	−0.20	-0.21
PANSS Total				−0.11	−0.15	-0.18
IMM	0.16	0.22	0.06	−0.14	0.20	0.12
VC	0.07	0.15	0.13	−0.06	0.05	-0.12
LAN	0.11	0.27	0.14	0.07	0.06	0.09
ATT	0.11	0.29*	0.28*	−0.03	0.14	0.18
DEM	-0.11	0.03	−0.17	−0.04	−0.04	-0.15
TOT	0.14	0.28*	0.31*	−0.01	0.08	-0.06
INT-C	0.51*	0.15	0.12	−0.13	0.04	0.02
INT-W	-0.05	−0.15	−0.29*	−0.02	0.10	0.12

Note: *SZ* schizophrenia patients, *HC* healthy controls, *PPI* prepulse inhibition, *PSC-PPI* perceived spatial co-location PPI, *PSS-PPI* perceived spatial separation PPI. CPZe: neuroleptic chlorpromazine-equivalent dosage. PANSS: positive and negative syndrome scale, *IMM* immediate memory score, *VC* visuospatial/constructional score, *LAN* language score, *ATT* attention score, *DEM* delayed memory score, *TOT* total composite score, *INT-C* color interference time, *INT-W* word interference time; * *p* < 0.05

In order to further discover the correlation between PPI and the symptoms severity of schizophrenia, we performed Pearson’s analysis using all potential confounding variables (age, gender, education, duration, recurrence times, onset, smoke amount and drug) as covariates [40]. We did not observe any significant association between the mean PPI and PANSS scores for all 75 patients and patients whose symptoms were classed as “remission” (total PANSS score < 60). However, in patients who were “non-remission” (total PANSS score ≥ 60) [41], we observed a significant correlation between the PSC-PPI, PSS-PPI with P1, P6, P7, PANSS Positive, N5, N7, G9 and PANSS Total, as well as a significant correlation between the PSS-PPI with PANSS Negative (see Table 6).

**Table 6:** Correlation coefficients between PPI and symptoms in patients (PANSS Total ≥ 60, Pearson’s *r, n = 35,df = 25)*

Variable	Startle	PSC-PPI	PSS-PPI
P1	-0.20	−0.39*	-0.52*
P2	0.02	−0.19	-0.31
P3	-0.09	−0.12	-0.17
P4	0.16	−0.29	-0.34
P5	0.05	−0.20	-0.24
P6	-0.18	−0.35*	-0.44*
P7	-0.01	−0.53*	-0.53*
PANSS Positive	−0.07	−0.37*	-0.47*
N1	0.06	−0.19	-0.31
N2	0.11	−0.07	-0.27
N3	0.05	−0.17	-0.28
N4	-0.04	−0.85	-0.21
N5	-0.05	−0.44*	-0.52*
N6	-0.02	−0.15	-0.30
N7	-0.10	−0.40*	-0.50*
PANSS Negative	−0.04	−0.27	-0.41*
G1	0.13	−0.21	-0.15
G2	0.25	−0.21	-0.21
G3	0.12	−0.33	-0.32
G4	0.13	−0.24	-0.32
G5	0.23	−0.11	-0.18
G6	-0.21	−0.27	-0.23
G7	-0.30	−0.21	-0.17
G8	0.15	−0.19	-0.28
G9	-0.25	−0.41*	-0.49*
G10	-0.13	−0.24	-0.08
G11	0.33	0.08	-0.23
G12	-0.17	0.09	-0.17
G13	-0.24	−0.22	-0.13
G14	0.18	−0.14	-0.11
G15	0.26	−0.13	-0.05
G16	-0.57*	−0.05	0.12
PANSS General	−0.02	−0.35	-0.24
PANSS Total	−0.02	−0.43*	−0.54*

Note: *PPI* prepulse inhibition, *PSC-PPI* perceived spatial co-location PPI, *PSS-PPI*, perceived spatial separation PPI. *PANSS* positive and negative syndrome scale. The control factors were gender, age, education, duration, recurrence times, onset age, cigarettes per day and neuroleptic chlorpromazine-equivalent dosage. No correlation was found between PPI and symptoms in total patients or in patients (PANSS Total < 60*).* * *p* < 0.05

Table 7 shows the independent correlates of performance on PSC-PPI and PSS-PPI in healthy controls and patients. In the healthy group, male and higher ATT scores contributed to better PSC-PPI and PSS-PPI, while high nicotine dependent and longer INT-W contributed to poorer PSS-PPI performance. In patients’ group, ATT and TOT scores accounted for 9% of the variation in PSC-PPI, and did not reach statistical significance. But higher education levels, ATT and TOT scores contributed to PSS-PPI, and recurrent episodes contributed to poorer PSS-PPI.

**Table 7:** Results of stepwise multiple regression analysis

PPI task	Predictor	Beta	S.E.	*t*	*p*
HC (*n* = 50)					
PSC-PPI	Gender	26.00	7.41	3.51	0.00
Adjusted R^2^ = 0.28;	ATT	0.56	0.20	2.79	0.01
F _(4,45)_ = 5.88; *p* < 0.001					
PSS-PPI	Gender	33.81	7.83	4.31	0.00
Adjusted R^2^ = 0.37;	Cigarettes per day	−0.95	0.43	−2.22	0.03
F _(6,43)_ = 5.88; *p* < 0.001	ATT	0.61	0.19	3.23	0.00
	INT-W	-0.77	0.32	−2.38	0.02
SZ (*n* = 75)					
PSC-PPI	ATT	1.17	0.43	2.27	0.01
Adjusted R^2^ = 0.09;	TOT	2.04	0.82	2.49	0.02
F _(8,66)_ = 1.96; *p* > 0.05					
PSS-PPI	Education	3.05	1.27	2.41	0.02
Adjusted R^2^ = 0.17;	Recurrence times	−3.10	1.25	−2.48	0.00
F _(8,66)_ = 2.85; *p* < 0.05	ATT	1.18	0.32	3.70	0.00
	TOT	1.86	0.48	3.86	0.00

Note: *S.E* standard error, *SZ* Schizophrenia patients, *HC* healthy controls. *PPI* prepulse inhibition, *PSC-PPI* perceived spatial co-location PPI, *PSS-PPI* perceived spatial separation PPI. *ATT* attention score, *TOT* total composite score, *INT-W* word interference time

## Discussion

To our knowledge, this is the first study employing a completely new PPI paradigm in a large cohort of Chinese schizophrenia patients to demonstrate reduced PPI and poor cognitive function. Moreover, there was a statistically significant deficit of PPI in patients compared with healthy controls and the effect sizes were large. Among patients with significant symptoms, the PPI deficits were negatively related to positive symptoms and thought disorders. Correlational and stepwise regression analyses further indicated that the underlying mechanisms of the new PPI were associated with attention modulation.

Most studies using “passive” paradigm showed PPI deficit at short lead intervals (≤ 60 ms), which relies mainly on automatic mechanisms [26]. Meanwhile, the “instructed” or active PPI deficits which can be manipulated volitionally were found in longer ISI condition [42], and the ISI 120 ms was the point that stimulus discrimination (prepulse and startle stimulus) and further attentional allocation occurred [43]. In our experiment, the perceived spatial co-location paradigm (PSC) of PPI in our study is like that of the “attention-to-prepulse” active paradigm, which employed subjective attention, however, the perceived spatial separation (PSS) paradigm involved not only subjective attention but also objective selective attention.

Previously published data confirmed that directing attention to the prepulse signal could enhance PPI [11, 44], and attention plays a key role in selecting relevant and ruling out irrelevant modalities, spatial locations, and task-related objects [45]. The reason for significantly enhanced PSS-PPI in controls may attribute to intact selective attention to the target stimulus, which is greatly impaired in patients [46]. So, this novel PSS-PPI paradigm which integrated measurements of sensory gating, attention and precedence effect, might achieve robust effect size for discriminating schizophrenia patients from healthy controls.

“Sensorimotor gating deficits” are hypothesized to contribute to sensory overload, interceptive stimuli and cognitive fragmentation, resulting in psychotic symptoms and cognitive deficits [47]. The results of this work indicate that the modified PPI was negatively correlated with symptom severity in group PANSS ≥60, and focused on thought disorder. Previous studies have shown strong associations between PPI deficits and thought disorder and distractibility. This relationship is most apparent when a specific, highly sensitive measure of PPI (novel PSS-PPI) is used. Thought disorder has been considered a hallmark feature of schizophrenia [48]. Besides one part of the positive symptom complex, factor analytic studies of symptom ratings suggested that disorganized thought is distinct from positive symptoms such as hallucinations and delusions. Moreover, thought disorder is different from positive and negative symptoms in development and course of the illness [49]. In this study, the PPI were correlated with P1, P6, P7, N5, N7 and G9, which included not only formal thought disorder but content of thought.

Previous researches have confirmed that the “attention-to-prepulse” PPI deficiency is more associated with the symptom severity in the schizophrenia spectrum and the correlates between symptoms [12]and PPI deficits in patients with schizophrenia cannot be detected in the passive-attention PPI paradigm [26]. From a Circular Inference models standpoint [50], the mechanism of the new PPI paradigm may involve a recognition (recognition of spatial separation of target–masker) evoked top-down modulation of information processing, which greatly impaired in schizophrenia patients [51]. Therefore, the new PPI paradigm may relate far more sophisticated thought elements rather than simple perception abnormal [52]. 

Regression analyses indicated that the modified PPI was typically related to attention scores, no matter in healthy controls or patients, especially for PSS-PPI. Stroop test has long been taken as a measure of perceptual switching, selective attention and cognitive inhibition [53, 54]. Accordingly, in our study, less INT-W might indicate better of PSS- PPI in control subjects but not in patients. These results agreed with the study of Bitsios and Giakoumaki [55], which indicated subjects with the best attentional selection in the Stroop task may be more prone to attentional selection of the prepulse and thus more likely to present with greater startle inhibition. However, patients partly lose cognitive inhibition [9, 56].

When interpreting the results of this study, several limitations must be considered. First, most patients enrolled in this study were chronic schizophrenia patients and did not manifest every possible symptom [57], which would not permit drawing assertive conclusion for heterogeneous schizophrenia. Therefore, future studies are needed to extend to first-episode, antipsychotic-naïve schizophrenia patients and to clinical high risk to validate the effectiveness. Second, this study included a higher male to female ratio. Although, the gender was controlled as a covariate, higher PPI levels have been reported in healthy males compared to healthy females [26]. In addition, PPI values may be influenced by the changes of menstrual period within the same female subjects [58]. Third, PPI levels in our study were relatively lower than those in previous studies [40], which may be due to parameter option of our PPI paradigm. However, we still obtained sufficient PPI levels and robust effect sizes as one study [59]. Finally, the cross-sectional study design, which precluded further examination stability and mediating variables over times. Ideally, such studies should employ larger samples to scrutinize trait versus state effects, illness phase specific, and the specific drug effect of the new PPI paradigm.

## Conclusion

In conclusion, this study developed a novel PPI paradigm firstly for human subjects. The modified PPI paradigm investigated not only sensorimotor gating but also attention function and correlated with symptom severity. The novel paradigm might be suitable for clinical study and application.

## Abbreviations

ATT: Attention score
AUC: Area under the ROC curve
DEM: Delayed memory score
EMG: Electromyography
HC: Healthy controls
IMM: Immediate memory score;
INT-C: Color interference time;
INT-W: Word interference time.
LAN: Language score
PANSS: Positive and negative syndrome scale
PPI: Prepulse inhibition
PSC-PPI: Perceived spatial co-location prepulse inhibition
PSS-PPI: Perceived spatial separation-induced prepulse inhibition
RBANS: Repeatable Battery for the Assessment of Neuropsychological Status
SCID: The structured clinical interview for DSM-IV
SZ: Schizophrenia patients
TOT: Total cognitive function score
VC: Visuospatial/constructional score
